# The burden of brain and central nervous system cancers in Asia from 1990 to 2019 and its predicted level in the next twenty-five years: Burden and prediction model of CNS cancers in Asia

**DOI:** 10.1186/s12889-023-17467-w

**Published:** 2023-12-16

**Authors:** Xin Liu, Lin-Can Cheng, Teng-Yu Gao, Jie Luo, Chao Zhang

**Affiliations:** 1 Center for Evidence-Based Medicine and Clinical Research, Taihe Hospital, Hubei University of Medicine, No.32, Renmin South Road, Shiyan, 442000 China; 2 Department of Neurosurgery, Center for Evidence-Based Medicine and Clinical Research, Taihe Hospital, Hubei University of Medicine, No. 32, Renmin South Road, Shiyan, 442000 China

**Keywords:** Global burden of disease, Brain and central nervous system cancers, Age-period-cohort, Asia, Cancer prediction

## Abstract

**Background:**

Primary brain and central nervous system cancer (collectively called CNS cancers) cause a significant burden to society. The purpose of this study was to evaluate the trends in the burden of CNS cancers from 1990 to 2019 and to predict the incidence and mortality rates and the corresponding numbers for the next 25 years to help countries to understand the trends in its incidence and mortality, and to make better adjustments or formulation of policies and allocation of resources thereby reducing the burden of the disease.

**Methods:**

The 2019 Global Burden of Disease Study provided incidence rates, death rates, and disability-adjusted life year (DALY) data in Asia from 1990 to 2019. To reflect the trends in the age-standardized incidence, mortality, and DALY rates, the estimated annual percentage change (EAPC) was determined. The Bayesian age-period cohort (BAPC) model was employed to predict the burden of CNS cancers in the next 25 years.

**Results:**

The incidence, death, and DALY rates of CNS cancers all increased from 1990 to 2019. The age-standardized incidence rate (ASIR) for CNS cancers increased from 9.89/100,000 in 1990 to 12.14/100,000 in 2019, with an EAPC of 0.69 (95% confidence interval (CI): 0.65, 0.73). The ASDR and the age-standardized DALY rate both decreased, with EAPCs of − 0.08 and − 0.52, respectively. Before 2005, the age-standardized DALY rate in East Asia was much greater in females than in males, while in Central Asia, the age-standardized death and DALY rates in males both increased sharply after 2000. In contrast to 1990, the caseload increased for the 55–70 years age group. The number of deaths decreased sharply among individuals aged younger than 20 years, especially in East Asia, accounting for only 5.41% of all deaths. The age group with the highest mortality rate was > 60 years, especially in Japan. The ASIR will continue to increase in Asia from 2020 to 2044, and the ASDR will gradually diminish. The incidence and number of deaths from CNS cancers in Asia are expected to increase over the next 25 years, especially among females.

**Conclusions:**

The study identified an increasing trend in morbidity, mortality and disability-adjusted life-years (DALYs), with differences in age-standardized morbidity rates for different population groups. In addition, it is noteworthy that the burden of disease (as measured by disability-adjusted life-years (DALYs)) is higher among women in Central Asia compared with other regions. ASIR will continue to increase over the next 25 years, with the increase in female cases and mortality expected to be more pronounced. This may need to be further substantiated by additional research, on the basis of which health authorities and policymakers can better utilize limited resources and develop appropriate policies and preventive measures.

**Supplementary Information:**

The online version contains supplementary material available at 10.1186/s12889-023-17467-w.

## Introduction

Primary brain and central nervous system cancers (collectively called CNS cancers) impose a considerable burden on society, as there were 33,000 incident cases of CNS cancer worldwide in 2016 [1, 2]. Due to the complex histology of central nervous system cancers, multiple types are listed in the World Health Organization International Classification of Neoplastic Diseases, of which approximately 30.2% of CNS cancers are malignant and 69.8% are benign [3–6]. Glioblastoma was the most common malignant CNS cancers (accounting for 14.6% of all CNS cancers and 48.3% of malignant CNS cancers), and meningioma was the most common nonmalignant CNS cancers (accounting for 37.6% of all CNS cancers and 53.3% of nonmalignant CNS cancers). Males were more likely to develop glioblastoma, whereas females were more likely to develop meningioma. CNS cancers (both malignant and nonmalignant) are the most common type of cancers among children aged 0–14 years, with an average annual age-adjusted incidence rate (AAAIR) of 5.74 per 100,000 population [6]. According to several studies, some individuals experience ocular symptoms before CNS symptoms. Bilateral involvement or vision loss is the most typical ocular symptom [7]. CNS cancers have an immeasurable burden on society as a whole, families, and individuals.

Some research showed that East Asia had the highest incidence of CNS cancers among males and females in 2016 (108,000 cases [98,000-122,000]), followed by Western Europe and South Asia. The top three countries with the most cases were China (106,207 cases), the United States (24,725 cases), and India (23,344 cases) [2]. As China and India are the leading countries in Asia, it is evident that the burden of CNS cancers in Asia cannot be ignored; however, there is little research specifically studying the incidence, death, and DALY rates of CNS cancers by sex and age in Asia. Previous attempts to estimate the prevalence of CNS cancers have only examined particular nations, regions, or annual estimates. Therefore, there is a necessity for systematic studies on the impact and prediction of CNS cancers burden in Asia to help us better understand the epidemiological characteristics of the disease and provide new insights into the development of CNS cancers prevention programs and their management and prevention in different countries.

Data from the GBD 2019 Study were used to quantify incidence, mortality, disability-adjusted life years (DALYs), and corresponding changes in CNS cancers, stratified by sex, age, and 48 countries in five subregions of Asia over the past 30 years. Then, we predicted the burden over the next 25 years, thus providing guidance for lowering the disease burden of CNS cancers in Asia and providing information to support government policy-making and public resource utilization control measures.

## Materials and methods

### Data resources

The GBD 2019 study (http://ghdx.healthdata.org/gbd-results-tool) provided information on the annual incidence, death, DALYs, respective age-standardized rate (ASR), and risk factors for brain and central nervous system cancers. The definition of CNS cancers in the GBD study was based on the tenth edition of the International Classification of Diseases (ICD). Briefly, CNS cancers include cancers coded as C70 (meningeal malignancies), C71 (brain malignancies), and C72 (malignancies of the spinal cord, cranial nerves, and other parts of the CNS) [8]. This study grouped Asia into five divisions (East Asia, West Asia, Central Asia, South Asia and Southeast Asia) and extracted social-demographic index (SDI) from 48 Asian nations to accurately examine the differences in incidence and mortality and DALYs and corresponding trends in Asia from 1990 to 2019. The SDI is a composite indicator of a country’s lag-distributed income per capita, average years of schooling, and the fertility rate in females under the age of 25 years. The 204 countries and territories were divided into five levels by SDI, high, high middle, middle, low middle, and low SDI was calculated using several social factors, including the fertility rate of the population aged < 25 years, the education level of the population aged > 15 years, and per capita income [9]. The GBD study data followed the Guidelines for Accurate and Transparent Health Estimation Reporting for Population Health Research (GATHER), but the study may have some limitations as we only used data from the GBD database, whose completeness and accuracy may be controversial, which may necessitate validation by further studies from other databases or official sources.

### Statistical analysis

Age-standardized incidence rate (ASID), age-standardized death rate (ASDR), age-standardized DALY rate, and the corresponding estimated annual percentage changes (EAPCs) were used to assess the trend in CNS cancers incidence and mortality.

To eliminate the influence of different age structures on disease outcomes when comparing the disease burden among different locations, the age-standardized rate (ASR) was employed. ASRs (per 100,000 population) were calculated based on the following formula: ASR= $\frac{\sum_{i=1}^A{a}_i{w}_i}{\sum_{i=1}^A{w}_i}\times 100,000$. *a*_*i*_ is the rate in the ith age group, and *w*_*i*_ is the GBD standard population number of the same age group [10]. Years of life lost (YLLs) and years lived with disability (YLDs) were added together to calculate the value of DALYs [11].

The concept of EAPC was introduced to reflect trends in ASR within specified time intervals and indicated time trends in age-standardized incidence, death, and DALY rates of CNS cancers: y = α + βx + ε, where y refers to ln (ASR), x represents the calendar year, and β determines the positive or negative trends in ASR [12]. The formula for calculating EAPC is EAPC = 100 × (exp(β)-1), and its 95% confidence interval (CI) was also obtained from the linear model. It has been shown that an upward trend in the ASR can be inferred if the EAPC value and its lower limit of 95% CI are both positive. In contrast, the ASR is thought to trend downwards if both the EAPC value and its upper boundary of the 95% CI are negative. Otherwise, ASR is regarded as stable [13]. We also created a similar scatter plot to visualize the link between EAPC, ASR, and SDI. We progressively refined the analysis of the distribution level of CNS cancers in Asia as a whole to the incidence, mortality, and DALY rates in the five major regions and individual countries, as well as analysing the relationship between EAPC and ASIR, ASDR, DALY rate, and SDI, and finally interpreting the predicted results for the next 25 years.

The *Nordpred* package in R was employed to perform an age-period-cohort (APC) analysis by sex, taking into account the changing rates and changing population structure, which has been fully demonstrated and acknowledged in prior studies, to predict the number of new cases and deaths from CNS cancers from 2020 to 2044 [14]. The absolute number of events that would occur if the rates remained stable (baseline reference), decreased by 1% per year (optimistic reference), and increased by 1% per year (pessimistic reference), based on the actual observed rates in 2019, were also calculated to make comparisons with the predicted results easier. The Bayesian APC model integrated nested Laplace approximation (INLA) in R was then used to conduct a sensitivity analysis utilizing the BAPC and INLA packages to confirm the stability of the prediction results [15].

The ρ indices Pearson’s correlation coefficient and *P* values were derived from Pearson’s correlation analysis.

### Uncertainty analysis

The uncertainty interval (UI) is calculated from the GBD database by multiple repeated sampling through the correlation matrix taking into account the differences between different calculation methods in different countries and the uncertainty of multiple filling of missing data values in different countries.

## Results

### Changes in the burden in Asia from 1990 to 2019

The number of cases of CNS cancers increased from 89,649.56 (95% UI, 70,036.58-123,551.00) in 1990 to 188,660.34 (95% UI, 142,442.95-214,510.36) in 2019 in all of Asia (Table 1). In contrast to the drastic increase in incident cases in the past 30 years, the ASIR was relatively steady, ranging from 9.89/100,000 persons (95% UI, 7.36–13.89) in 1990 to 12.14/100,000 persons (95% UI, 8.74–14.29) in 2019 (Table 1). The number of annual deaths increased from 70,769.80 (95% UI, 55,089.26-97,707.31) to 129,519.80 (95% UI, 96,994.74-146,915.01) in Asia, and the ASDR decreased slightly with an EAPC of − 0.08 (95% CI, from − 0.13 to − 0.03), decreasing from 2.76/100,000 persons (95% UI, 2.21–3.70) in 1990 to 2.75/100,000 persons (95% UI, 2.06–3.10) in 2019 (Table 2). The number of DALYs in Asia increased from 3,502,849.91 in 1990 to 4,776,406.31 in 2019 (95% UI, 3,638,971.48-5,463,357.90), and the age-standardized DALY rate decreased from 115/100,000 people (95% UI, 86–163) in 1990 to 103/100,000 people (95% UI, 78–118) in 2019 (Table 3). In the meantime, we also observe that countries with higher SDI have lower ASDR with age-standardized DALY rates (Fig. 2C, D), which may be related to the fact that countries with high SDI levels can provide better healthcare conditions and resources, which reduces the mortality rate of the disease and the adjusted year of disability illness.

**Table 1:** The incident cases and ASIR in 1990 and 2019 and their temporal trends

Regions	1990		2019		1990–2019 EAPC No.(95% CI)
	Incident cases No.(95% UI)	ASIR per 100,000 No.(95% UI)	Incident cases No.(95% UI)	ASIR per 100,000 No.(95% UI)	
**Divisions**					
**Asia**	89,649.56 (70,036.58,123,551.00)	9.89 (7.36,13.89)	188,660.34 (142,422.95,214,510.36)	12.14 (8.74,14.29)	0.69 (0.65,0.73)
Female	38,326.03 (26,394.67,54,648.28)	2.87 (2.08,3.98)	88,790.47 (66,821.31,105,302.86)	3.77 (2.84,4.45)	0.88 (0.82,0.94)
Male	51,323.52 (34,242.01,77,739.43)	3.73 (2.6,5.42)	99,869.87 (66,151.92,122,393.92)	4.33 (2.85,5.26)	0.54 (0.52,0.57)
**Central Asia**	1942.89 (1732,2667.46)	9.68 (8.58,13.25)	4449.66 (3027.92,5334.47)	15.03 (9.9,17.97)	1.88 (1.69,2.06)
Female	867.23 (752.02,1235.91)	2.66 (2.33,3.76)	1853.28 (1188.55,2246.27)	3.98 (2.57,4.8)	1.72 (1.54,1.90)
Male	1075.65 (944.47,1491.55)	3.82 (3.36,5.21)	2596.37 (1658.79,3101.82)	6.09 (3.93,7.24)	1.99 (1.79,2.19)
**East Asia**	47,262.86 (36,377.85,63,147.05)	13.29 (9.41,18.52)	96,818.14 (75,147.63,116,192.36)	16.93 (12.17,21.5)	0.74 (0.67,0.81)
Female	21,409.15 (14,207.92,28,857.98)	4.06 (2.74,5.49)	48,598.53 (35,486.66,62,888.7)	5.73 (4.26,7.6)	1.04 (0.95,1.14)
Male	25,853.71 (17,282.82,38,674.09)	4.81 (3.21,7.14)	48,219.61 (31,357.32,62,919.7)	5.58 (3.6,7.22)	0.48 (0.43,0.53)
**South Asia**	19,935.9 (14,074.49,30,847.61)	6.08 (4.17,9.43)	37,190.71 (28,418.59,44,752.36)	6.84 (4.97,8.52)	0.27 (0.18,0.35)
Female	6879.08 (4024.14,13,059.84)	1.46 (0.92,2.48)	15,061.66 (11,721.53,18,206.88)	1.87 (1.46,2.26)	0.71 (0.60,0.82)
Male	13,056.82 (7845.65,21,148.62)	2.57 (1.69,3.95)	22,129.06 (14,856.18,29,025.43)	2.68 (1.78,3.52)	0.05 (−0.03,0.13)
**Southeast Asia**	8372.21 (5974.05,11,979.36)	6.62 (4.52,9.44)	16,552.84 (10,921.37,19,910.91)	7.65 (4.77,9.53)	0.56 (0.52,0.59)
Female	3827.36 (2381.18,6211.86)	2.00 (1.31,3.04)	7835.91 (5117.86,9716.94)	2.34 (1.53,2.91)	0.59 (0.53,0.64)
Male	4544.85 (2762.34,7013.12)	2.42 (1.58,3.45)	8716.93 (4907.67,11,352.95)	2.76 (1.57,3.56)	0.54 (0.50,0.58)
**West Asia**	464.49 (276.31,713.17)	4.73 (3.47,6.99)	1192.81 (694.47,1604.27)	6.01 (4.05,7.9)	0.98 (1.04,0.91)
Female	193.15 (98.13,375.2)	4.03 (2.59,6.81)	528.44 (285.82,764.12)	5.41 (3.34,7.77)	1.21 (1.28,1.13)
Male	271.34 (138.57,138.57)	5.41 (3.52,8.49)	664.38 (342.66,943.39)	6.64 (4.02,9.12)	0.83 (0.9,0.76)
**Countries**					
Afghanistan	501.7 (234.79,1143.16)	14.81 (6.42,35.6)	1264.06 (693.54,2275.52)	15.27 (7.93,31.32)	0.17 (0.07,0.27)
Armenia	221.06 (181.21,273.23)	20.85 (15.97,26.39)	248.13 (192.81,299.35)	20.59 (15.66,25.41)	0.1 (−0.07,0.26)
Azerbaijan	307.26 (255.92,376.94)	14.46 (11.03,18.28)	540.35 (379.24,685.32)	15.25 (9.65,20.56)	0.07 (−0.03,0.16)
Bahrain	10.17 (7.39,13.22)	9.72 (6.4,13.75)	46.6 (28.48,61.49)	10.69 (6.74,14.09)	0.51 (0.34,0.67)
Bangladesh	2231.03 (1256.51,3755.84)	6.73 (3.94,11.16)	2919.12 (1887.86,3972.58)	5.99 (3.57,8.74)	−0.52 (− 0.58,-0.46)
Bhutan	10.84 (4.91,20.87)	6.24 (3.33,11.64)	15.48 (9.4,23.29)	7 (4.11,10.95)	0.2 (0.09,0.32)
Brunei Darussalam	9.62 (7.18,15.66)	16.86 (12.31,26.58)	23.42 (18.27,28.79)	20.25 (14.25,25.19)	0.86 (0.72,1)
Cambodia	211.9 (109.11,401.59)	7.92 (3.99,14.53)	429.03 (269.2584.32)	9.07 (5.3,13.45)	0.47 (0.41,0.54)
China	45,846.92 (35,180.53,61,348.34)	13.38 (9.44,18.72)	94,685.54 (73,400.6114091.97)	17.16 (12.27,21.87)	0.76 (0.69,0.83)
Cyprus	41.38 (34.92,58.7)	15.42 (12.21,22.83)	130.49 (87.17,158.02)	23.27 (14.74,29.42)	1.81 (1.65,1.96)
Democratic People’s Republic of Korea	836.4 (523.06,1241.18)	12.71 (7.28,20.01)	1146.37 (762.19,1511.78)	11.6 (7.38,15.85)	−0.2 (−0.28,-0.12)
Georgia	388.98 (345.32,528.29)	17.27 (14.71,23.89)	865.46 (659.38,1094.08)	19.03 (13.85,25.24)	2.49 (2.16,2.82)
India	14,958.45 (10,694.43,24,147.94)	5.95 (4.03,9.34)	28,102.76 (21,991.64,34,272.78)	6.64 (4.85,8.4)	0.19 (0.08,0.29)
Indonesia	3239.49 (2239.96,4872.35)	6.39 (4.2,9.65)	5658.45 (3960.41,7306.55)	7.04 (4.58,9.56)	0.35 (0.27,0.43)
Iran (Islamic Republic of)	2522.54 (1433.78,3394.15)	16.78 (9.11,23.8)	5810.58 (2942.35,7045.75)	21.83 (10.39,27.45)	0.83 (0.55,1.12)
Iraq	768.52 (541.03,1389.76)	17.58 (11.5,32.18)	2797.04 (2019.19,3628.3)	25.65 (17.87,33.89)	1.51 (1.21,1.82)
Israel	287.46 (251.45,407.45)	18.03 (15.56,26.05)	930.42 (566.74,1239.22)	27.3 (15.98,37.18)	1.27 (1.05,1.5)
Japan	4321.53 (3891.11,5995.49)	9.3 (8.21,13.27)	11,338.76 (5517.85,14,811.75)	15.45 (7.59,20.32)	2.28 (1.94,2.62)
Jordan	85.77 (68.4118.57)	10.41 (7.67,15.27)	409.43 (295.47,507.84)	13.1 (8.79,17.13)	0.88 (0.83,0.92)
Kazakhstan	206.29 (154.03,364.53)	4.05 (2.89,7.62)	653.25 (324.33,849.85)	10.6 (4.92,14.01)	4.59 (3.89,5.3)
Kuwait	36.07 (30.69,46.8)	8.29 (6.91,11.52)	144.33 (101.36,180.84)	11.79 (7.98,15.44)	1.86 (1.65,2.08)
Kyrgyzstan	62.87 (48.93,111.68)	4.95 (3.88,8.57)	163.34 (96.74,203.98)	8.53 (4.78,10.9)	1.91 (1.74,2.08)
Lao People’s Democratic Republic	105.85 (48.59,221.34)	9.12 (4.09,19.4)	157.67 (100.77,217.95)	8.11 (4.83,11.95)	−0.47 (−0.52,-0.43)
Lebanon	118.7 (91.17,161)	12.87 (9.27,18.28)	350.15 (251.65,447.06)	20.23 (13.77,27.07)	1.93 (1.7,2.15)
Malaysia	304.39 (188.79,392.17)	6.62 (3.94,8.94)	684 (437.83,921.73)	6.82 (4.2,9.42)	−0.01 (−0.23,0.22)
Maldives	5.04 (2.94,10.25)	9.33 (5.33,18.58)	11.66 (8.98,15.83)	8.38 (6.17,11.14)	−0.49 (− 0.69,-0.3)
Mongolia	31.62 (21.31,62.31)	6.06 (4.07,11.91)	112.74 (75.72,150.44)	11.46 (6.92,15.62)	2.88 (2.46,3.31)
Myanmar	945.99 (496.73,1893.36)	8.1 (3.99,16.19)	1358.43 (927.65,1816.29)	7.97 (5.11,10.91)	−0.18 (−0.27,-0.08)
Nepal	353.2 (227.28,588.67)	5.78 (3.68,9.8)	514.05 (361.36,660.38)	5.8 (3.78,8.03)	0.13 (−0.02,0.27)
Oman	27.84 (20.81,40.18)	6.45 (4.42,10.06)	123.45 (71.6161.47)	11.09 (6.47,14.9)	2.27 (2,2.53)
Pakistan	2382.38 (1629.64,3664.51)	6.41 (4.68,9.09)	5639.3 (3968.01,7307.1)	8.24 (5.66,10.92)	0.91 (0.83,0.99)
Palestine	117.53 (85.56,173.1)	25.99 (17.42,39.9)	323.99 (248.54,394.63)	28.43 (19.78,34.92)	0.26 (0.06,0.45)
Philippines	1287.24 (1056.34,1691.52)	8.05 (6.26,10.25)	2432.19 (1849.86,2994.66)	7.55 (5.35,9.79)	−0.32 (− 0.53,-0.11)
Qatar	8.94 (6.37,15.43)	11.64 (8.08,18.82)	96.77 (63.04,160.63)	16.29 (11.28,23.95)	1.38 (1.22,1.54)
Republic of Korea	1513.02 (1080.29,2004.28)	12.54 (8.35,16.64)	4805.51 (2898.99,5975.96)	20.2 (11.77,26.11)	1.5 (1.34,1.66)
Saudi Arabia	178.66 (97.89,402.82)	5.14 (2.63,12.35)	1295.78 (944.39,1961.54)	12.28 (8.57,19.24)	3.46 (3.08,3.84)
Singapore	76.96 (67.49,111.7)	9 (7.51,13.33)	308.78 (169.77,412.56)	13.81 (6.98,19.02)	1.8 (1.54,2.06)
Sri Lanka	221.7 (183.07,327.5)	4.79 (3.9,7.29)	631.25 (336,891.77)	8 (4.05,11.51)	2.73 (2.37,3.09)
Syrian Arab Republic	413.47 (304.89,633.43)	15.17 (9.98,21.37)	803.87 (567.54,1083.3)	18.16 (12.1,24.59)	0.6 (0.47,0.73)
Taiwan (Province of China)	579.54 (501.13,721.49)	9.2 (7.47,11.7)	986.22 (611.63,1332.28)	10.79 (6.17,14.7)	0.29 (0.16,0.42)
Tajikistan	220.86 (174.75,310.73)	16.6 (11.85,21.4)	465.58 (305.9600.37)	18.34 (11.35,23.78)	0.13 (−0.06,0.31)
Thailand	1335.28 (827.59,1630.86)	8.38 (4.97,10.88)	2994.62 (1312.89,4407.61)	10.48 (4.5,15.2)	0.69 (0.51,0.86)
Timor-Leste	12.58 (6.91,24.98)	6.36 (3.67,11.19)	26.86 (14.58,37.89)	7.58 (4.2,10.94)	0.65 (0.41,0.89)
Turkey	2886.39 (1339.89,4371.33)	18.11 (7.83,30.69)	6355.15 (2999.03,8787)	22.82 (10.19,31.56)	1.2 (0.76,1.64)
Turkmenistan	46.71 (24.22,134.59)	4.57 (2.42,12.85)	205.83 (138.7266.79)	12.92 (8.14,16.87)	4.45 (3.69,5.21)
United Arab Emirates	64.52 (45.22,91.05)	15.22 (9.88,25.41)	478.58 (274.75,703.53)	16.56 (9.79,23.65)	0.24 (0.15,0.33)
Uzbekistan	647.13 (567.79,812.26)	11.27 (9.22,15.19)	1794.57 (1178.42,2225.45)	17.7 (11.68,21.98)	1.84 (1.71,1.97)
Viet Nam	673.95 (505.65,914.95)	3.88 (2.84,5.33)	2110.95 (1285.42770.35)	6.49 (3.8,8.56)	2.36 (2.13,2.58)
Yemen	302.81 (149.55,624.77)	9.25 (4.48,19.23)	841.62 (503.06,1210.56)	11.65 (6.65,18.79)	1.07 (0.95,1.19)

*ASIR* Age-standardized incidence rate, *CI* Confidence interval, *EAPC* Estimated annual percentage changes, *UI* Uncertainty interval

**Table 2:** The death cases and ASDR in 1990 and 2019 and their temporal trends

Regions	1990		2019		1990–2019 EAPC No.(95% CI)
	Death cases No.(95% UI)	ASDR per 100,000 No.(95% UI)	Death cases No.(95% UI)	ASDR per 100,000 No.(95% UI)	
**Divisions**					
**Asia**	70,869.8 (55,089.26,97,707.31)	2.76 (2.21,3.7)	129,519.8 (96,994.74,146,915.01)	2.75 (2.06,3.1)	−0.08 (−0.13,-0.03)
Female	29,666.46 (20,731.36,42,378.13)	2.34 (1.71,3.24)	56,375.09 (41,207.37,66,805.93)	2.35 (1.7,2.78)	−0.11 (− 0.17,− 0.05)
Male	41,203.35 (28,029.93,61,555.62)	3.18 (2.26,4.69)	73,144.71 (47,240.14,89,396.9)	3.16 (2.03,3.85)	-0.05 (−0.09,0.01)
**Central Asia**	1630.93 (1464.22204.36)	2.81 (2.55,3.73)	3751.78 (2550.84495.93)	4.31 (2.95,5.13)	1.85 (1.65,2.05)
Female	726.89 (633.35,1035.34)	2.32 (2.02,3.27)	1550.25 (993.77,1879.5)	3.4 (2.19,4.1)	1.67 (1.48,1.87)
Male	904.04 (798.22,1244.24)	3.42 (2.96,4.66)	2201.53 (1401.62,2625.56)	5.37 (3.49,6.38)	1.97 (1.76,2.17)
**East Asia**	39,003.96 (29,980,51,606.41)	3.84 (3.02,5.04)	65,212.61 (48,947.82,78,579.95)	3.47 (2.61,4.15)	−0.49 (−0.58,-0.39)
Female	17,186.71 (11,996.68,22,950.67)	3.41 (2.42,4.53)	28,658.54 (20,861.14,36,973.72)	2.99 (2.19,3.82)	−0.7 (− 0.82,-0.58)
Male	21,817.25 (14,947.35,32,724)	4.31 (3.01,6.4)	36,554.07 (22,757.92,48,392.41)	4.01 (2.53,5.24)	−0.31 (− 0.39,-0.23)
**South Asia**	16,209.12 (11,622.99,24,770.81)	1.82 (1.41,2.61)	30,747.63 (23,852.64,36,824.31)	1.95 (1.51,2.34)	0.13 (0.04,0.21)
Female	5581.34 (3364.38,10,351.59)	1.3 (0.84,2.12)	12,532.45 (9572.915351.38)	1.61 (1.22,1.97)	0.62 (0.52,0.72)
Male	10,627.78 (6579.49,16,654.05)	2.29 (1.54,3.42)	18,215.19 (12,505.95,23,770.62)	2.29 (1.56,2.99)	−0.12 (−0.21,-0.03)
**Southeast Asia**	6976.67 (5057.04,9694.3)	1.99 (1.51,2.6)	14,172.35 (9387.67,17,247.45)	2.24 (1.49,2.7)	0.48 (0.43,0.53)
Female	3191.35 (2051.95031.27)	1.8 (1.21,2.66)	6705.49 (4228.15,8266.69)	2.04 (1.28,2.51)	0.47 (0.4,0.54)
Male	3785.32 (2352.75,5719.27)	2.19 (1.46,3.05)	7466.87 (4113.87,9708.53)	2.45 (1.36,3.16)	0.49 (0.45,0.54)
**West Asia**	357.48 (216.19,536.71)	3.98 (2.89,5.87)	761.08 (438.87,1024.03)	3.99 (2.68,5.24)	0.09 (0.13,0.05)
Female	148.53 (76.96,279.85)	3.36 (2.13,5.65)	340.75 (182.9493.51)	3.56 (2.19,5.13)	0.33 (0.38,0.29)
Male	208.95 (108.71,343.64)	4.61 (2.97,7.23)	420.33 (215.46,591.25)	4.45 (2.68,6.06)	−0.05 (−0.01,-0.09)
**Countries**					
Afghanistan	422.35 (208.91,944.43)	4.52 (2.49,9.4)	1014.01 (569.36,1847.56)	4.66 (2.84,8.48)	0.89 (0.41,1.37)
Armenia	190.84 (158.43,233.2)	6.2 (5.13,7.55)	220.36 (167.37,266.58)	5.8 (4.5,7)	−0.59 (−1.49,0.31)
Azerbaijan	262.16 (216.97,317.53)	4.24 (3.46,5.1)	466.79 (327.26,589.61)	4.39 (3.16,5.51)	0.07 (−0.42,0.56)
Bahrain	7.68 (5.47,10.02)	2.88 (2,3.99)	25.55 (15.88,34.14)	2.14 (1.44,2.75)	−2.27 (−2.63,-1.9)
Bangladesh	1768.06 (1038.08,2874.07)	2.01 (1.36,2.88)	2449.8 (1560.43347.11)	1.71 (1.09,2.34)	−1.12 (−1.22,-1.01)
Bhutan	8.58 (4.2,15.85)	1.86 (1.13,2.99)	12.7 (7.78,18.95)	1.99 (1.25,2.89)	0.13 (−0.08,0.35)
Brunei Darussalam	6.18 (4.65,9.91)	3.97 (3.08,5.91)	13.59 (10.5,16.54)	3.97 (2.97,4.71)	1.14 (0.57,1.72)
Cambodia	174.69 (93.48,321.25)	2.4 (1.48,3.91)	373.08 (232.71,509.74)	2.75 (1.72,3.75)	1.22 (1.08,1.36)
China	37,966.01 (29,102.47,50,213.17)	3.88 (3.04,5.11)	63,527.03 (47,792.73,76,948.19)	3.5 (2.62,4.21)	−1.78 (−2.15,-1.4)
Cyprus	30.11 (25.14,42.11)	3.71 (3.12,5.23)	65.91 (41.53,80.41)	3.64 (2.34,4.41)	0.33 (0.02,0.64)
Democratic People’s Republic of Korea	665.1 (435.37,947.72)	3.52 (2.36,4.89)	1027.96 (682.87,1346.05)	3.37 (2.24,4.36)	−0.28 (−0.4,-0.15)
Georgia	174.35 (141.03,252.91)	2.91 (2.35,4.26)	244.92 (136.63,311.57)	4.89 (2.81,6.21)	10.49 (8.94,12.06)
India	12,367.31 (9007.79,19,434.27)	1.79 (1.41,2.67)	23,740.26 (18,620.04,28,920.37)	1.91 (1.49,2.32)	0.14 (−0.05,0.33)
Indonesia	2709.37 (1925.81,3967.33)	1.94 (1.44,2.64)	4987.9 (3396.78,6395.05)	2.15 (1.48,2.71)	0.81 (0.61,1)
Iran (Islamic Republic of)	1768.78 (1038.42266.84)	4.63 (2.81,5.94)	3493.94 (1750.98,4173.06)	4.57 (2.26,5.47)	0.18 (−1.16,1.54)
Iraq	584.67 (413.91032.38)	5.07 (3.58,8.6)	1842.49 (1330.05,2362.3)	6.38 (4.69,8.17)	5.78 (4.23,7.35)
Israel	195.87 (173.93,276.59)	4.1 (3.64,5.73)	513.79 (313.51,591.35)	4.77 (2.96,5.47)	1.52 (0.3,2.75)
Japan	1454.62 (1333.32,2014.36)	0.99 (0.91,1.38)	3222.69 (1554.09,3889.15)	1.36 (0.69,1.6)	1.73 (1.5,1.96)
Jordan	63.16 (49.63,87.09)	3.01 (2.4,4.25)	228.27 (162.41,282.34)	2.84 (2.04,3.53)	−0.55 (−0.78,-0.32)
Kazakhstan	170.29 (123.77,310.12)	1.15 (0.82,2.11)	541.82 (266.86,706.22)	2.91 (1.43,3.79)	9.48 (7.84,11.14)
Kuwait	19.41 (16.86,25.3)	1.87 (1.63,2.51)	59.19 (41.67,73.98)	1.89 (1.34,2.38)	1 (0.43,1.57)
Kyrgyzstan	53.37 (42.08,92.52)	1.46 (1.17,2.44)	138.84 (79.84,174.52)	2.49 (1.42,3.15)	4.03 (3.68,4.39)
Lao People’s Democratic Republic	88 (42.49,179)	2.76 (1.56,5.09)	135.86 (86.59,186.83)	2.49 (1.58,3.38)	−1.11 (−1.2,-1.02)
Lebanon	92.26 (71.91,124.94)	3.56 (2.82,4.77)	168.34 (121.83,220)	3.23 (2.33,4.21)	−0.51 (− 0.89,-0.14)
Malaysia	248 (156.34,317.51)	1.96 (1.3,2.44)	534.32 (349.64,710.05)	1.84 (1.23,2.42)	−0.61 (− 0.95,-0.26)
Maldives	4.08 (2.41,7.86)	2.92 (1.92,4.99)	8.2 (6.22,10.81)	2.21 (1.65,2.81)	−2.88 (−3.24,-2.52)
Mongolia	27.07 (18.31,52.16)	1.86 (1.27,3.36)	100.34 (65.48,134.81)	3.51 (2.13,4.7)	7.96 (6.75,9.18)
Myanmar	793.7 (422.49,1477.39)	2.44 (1.43,4.3)	1185.45 (813.18,1578.22)	2.37 (1.63,3.13)	−0.44 (−0.69,-0.19)
Nepal	280.74 (186.85,455.33)	1.71 (1.27,2.5)	440.32 (311.07,569.92)	1.72 (1.23,2.22)	0.26 (0.05,0.47)
Oman	19.64 (14.63,28.77)	1.83 (1.32,2.77)	54.32 (32.47,70.09)	2.13 (1.4,2.64)	2.13 (1.52,2.75)
Pakistan	1784.45 (1273.11,2595.61)	1.88 (1.44,2.54)	4104.57 (2847.05,5343.6)	2.27 (1.6,2.98)	1.39 (1.16,1.63)
Palestine	88.25 (65.93,129.66)	7.53 (5.58,10.81)	209.98 (158.82,250.7)	7.24 (5.24,8.68)	−1.07 (−2.04,-0.08)
Philippines	1067.82 (879.72,1356.65)	2.45 (2.06,2.93)	2073.06 (1561.21,2527.92)	2.27 (1.69,2.76)	−0.89 (−1.35,-0.42)
Qatar	6.26 (4.49,10.65)	3.38 (2.63,5.23)	36.63 (24.23,61.63)	2.81 (2.06,3.99)	−1.8 (−2.18,-1.42)
Republic of Korea	974.23 (689.21,1273.73)	2.74 (1.98,3.47)	1479.34 (923.78,1750.51)	1.91 (1.21,2.25)	−3.57 (−4.19,-2.95)
Saudi Arabia	152.55 (82.09,344.95)	1.71 (0.91,3.94)	596.75 (436.6915.32)	2.27 (1.68,3.54)	2.75 (1.64,3.87)
Singapore	37.86 (33.67,55.21)	1.5 (1.33,2.17)	96.92 (52.49,114.97)	1.37 (0.74,1.63)	−0.31 (− 0.63,0)
Sri Lanka	186.76 (155,274.05)	1.46 (1.22,2.08)	524.53 (270.21,748.94)	2.18 (1.12,3.12)	4.54 (3.73,5.35)
Syrian Arab Republic	325.81 (239.45,469.54)	4.58 (3.25,5.92)	566.3 (388.45,767.87)	4.46 (3.02,5.99)	−1.06 (−1.56,-0.57)
Taiwan (Province of China)	372.86 (324.43,456.81)	2.09 (1.82,2.56)	657.63 (400.5886.89)	2 (1.23,2.67)	−1.05 (− 1.4,-0.7)
Tajikistan	188.43 (149.36,246.78)	5.05 (3.75,6.06)	396.81 (257.23,511.98)	5.58 (3.56,7.17)	0.93 (0.04,1.83)
Thailand	1092.99 (687.93,1322.85)	2.45 (1.55,2.95)	2517.89 (1064.55,3739.99)	2.74 (1.19,4.02)	0.92 (0.44,1.4)
Timor-Leste	10.25 (6.04,18.8)	1.96 (1.35,3.01)	23.01 (13.12,32.2)	2.33 (1.38,3.24)	1.37 (0.95,1.79)
Turkey	2340.16 (1117.34,3454.82)	5.31 (2.62,7.84)	4074.5 (1832.75,5723.9)	4.73 (2.15,6.6)	−0.58 (−2.43,1.31)
Turkmenistan	38.12 (20,107.09)	1.35 (0.73,3.58)	172.76 (114.89,223.46)	3.71 (2.43,4.78)	11.41 (9.22,13.65)
United Arab Emirates	46.73 (32.81,66.85)	4.5 (3.02,7.12)	314.22 (180.2465.15)	4.2 (2.62,5.75)	−1.02 (− 1.38,-0.66)
Uzbekistan	526.34 (456.86,661.02)	3.25 (2.68,4.16)	1469.17 (975.05,1818.43)	5.06 (3.52,6.18)	7.92 (7.25,8.59)
Viet Nam	577.67 (439.61,776.56)	1.18 (0.92,1.56)	1760.26 (1069.98,2320.37)	1.81 (1.11,2.35)	3.06 (2.72,3.39)
Yemen	238.13 (125.32,474.86)	2.81 (1.74,5.11)	652.9 (397.99,949.52)	3.42 (2.19,5.06)	2.71 (2.4,3.02)

*ASDR* Age-standardized death rate, *CI* Confidence interval, *EAPC* Estimated annual percentage changes, *UI* Uncertainty interval

**Table 3:** The DALYs and age-standardized DALY rate in 1990 and 2019 and their temporal trends

Regions	1990		2019		1990–2019 EAPC No.(95% CI)
	DALYs No.(95% UI)	Age-standardized DALY rate per 100,000 No.(95% UI)	DALYs No.(95% UI)	Age-standardized DALY rate per 100,000 No.(95% UI)	
**Divisions**					
**Asia**	3,502,849.91 (2,531,323.74,5,105,631.75)	115.03 (86.22,162.92)	4,776,406.34 (3,638,971.48,5,463,357.9)	102.8 (78.16,117.75)	−0.52 (− 0.59,-0.46)
Female	1,434,010.77 (913,361.44,2,208,875.41)	96.78 (64.28,144.8)	2,008,497.11 (1,520,951.91,2,385,839.72)	87.08 (66.3103.19)	−0.59 (− 0.69,-0.49)
Male	2,068,839.14 (1,302,184.63,3,228,669.5)	132.55 (87.12,199.35)	2,767,909.22 (1,817,841.35,3,404,543.94)	118.2 (77.56,144.7)	−0.46 (− 0.51,-0.42)
**Central Asia**	79,029.43 (68,763.69,110,071.63)	120.6 (107.31,164.36)	162,291.28 (109,999.98,194,835.32)	173.86 (118.7208.26)	1.56 (1.38,1.74)
Female	34,883 (29,600.52,50,503.45)	101.53 (88.04,143.92)	65,905.38 (42,465.48,80,161.5)	138.16 (89.51,167.85)	1.36 (1.18,1.54)
Male	44,146.43 (38,059.94,64,554.64)	142.34 (125.45,196.74)	96,385.9 (61,969.92,115,616.76)	212.87 (137.58,254.27)	1.71 (1.51,1.9)
**East Asia**	1,817,549.89 (1,318,939.76,2,496,935.21)	159.74 (116.79,217.95)	2,110,763.86 (1,624,280.72585838.4)	125.41 (95.89,152.69)	−1.11 (− 1.25,-0.98)
Female	784,957.4 (507,646.25,1,072,686.23)	142.49 (92.84,194.87)	900,249.17 (673,719.91,1,182,735.92)	108.7 (80.51,142.84)	−1.35 (− 1.54,-1.16)
Male	1,032,592.49 (675,435.66,1,605,025.47)	176.62 (116.77,273.66)	1,210,514.7 (772,865.22,1,596,506.71)	142.09 (89.73,186.33)	−0.93 (−1.02,-0.83)
**South Asia**	935,871.47 (607,953.45,1,518,962.03)	82.06 (58.04,126.21)	1,383,154.56 (1,081,666.99,1,662,443.04)	81.08 (63.4,97.76)	−0.17 (− 0.26,-0.09)
Female	326,539.84 (176,873.91,675,086.98)	59.06 (35.13,111.04)	541,656.41 (422,887.68,662,721.45)	64.57 (50.56,78.43)	0.13 (0.03,0.24)
Male	609,331.63 (342,372.13,1,012,990.3)	103.11 (63.22,162.79)	841,498.15 (575,663.06,1,081,983.03)	96.9 (65.99,124.72)	−0.31 (−0.39,-0.23)
**Southeast Asia**	352,645.62 (234,131.47,535,670.32)	81.48 (57.45,116.47)	552,007.82 (379,458.87,669,066.56)	82.44 (57.08,99.53)	0.08 (0.05,0.11)
Female	155,109.91 (91,555.89,271,689.27)	71.94 (44.86,117.87)	251,520.13 (164,225.28,312,149.65)	74.22 (49.04,91.68)	0.11 (0.05,0.18)
Male	197,535.71 (109,760.96,329,724.13)	91.16 (55.49,140.86)	300,487.69 (172,848.95,393,313.84)	90.88 (52.74,118.46)	0.05 (0.03,0.08)
**West Asia**	17,070.05 (9672.43,26,899.92)	146.81 (105.12,221.64)	29,169.61 (17,354.14,39,423.73)	138.45 (93.64,182.1)	−0.13 (− 0.09,-0.16)
Female	7051.08 (3375,14,530.04)	124.22 (77.45,218.05)	12,900.48 (7072.91,19,353.14)	123.55 (76.82,181.06)	0.1 (0.14,0.06)
Male	10,018.97 (4866.76,17,417.39)	168.68 (106.92,269.96)	16,269.14 (8550.94,23,226.95)	153.71 (93.49,211.07)	−0.26 (− 0.23,-0.3)
**Countries**					
Afghanistan	21,579.97 (8799.57,50,914.48)	186.91 (89.39,431.5)	54,529.27 (29,084.11,95,998.72)	176.03 (99.65,323.91)	−0.11 (− 0.24,0.02)
Armenia	8293.06 (6677.54,10,428.99)	249.06 (204.07,310.16)	7508 (5907.47,9022.17)	219.3 (174.24,263.6)	−0.26 (− 0.44,-0.08)
Azerbaijan	12,414.55 (10,246.53,15,993.46)	180.33 (149.67,222.14)	19,153.66 (13,499.01,24,307.15)	177.28 (127.45,224.7)	−0.24 (− 0.36,-0.13)
Bahrain	356.87 (262.47,459.48)	97.32 (68.61,127.99)	1022.11 (617.93,1351.9)	72.99 (46.72,93.35)	−0.9 (−1,-0.81)
Bangladesh	110,846.3 (57,856,197,112.85)	93.12 (55.89,149.03)	111,974.7 (74,206.4150290.76)	74.26 (48.9,99.6)	−0.82 (− 0.88,-0.77)
Bhutan	529.06 (216.15,1074.06)	83.88 (41.96,152.89)	590.02 (348.24,909.54)	85.47 (51.07,130.05)	−0.19 (− 0.36,-0.02)
Brunei Darussalam	316.72 (231.29,531.19)	146.81 (110.26,233.15)	562.4 (451.24,713.87)	139.73 (109.47,172.61)	0.08 (−0.09,0.25)
Cambodia	9690.45 (4561.67,19,425.57)	98.75 (53.96,176.28)	15,587.21 (9769.58,21,119.14)	101.14 (63.89,137.14)	0.06 (0,0.13)
China	1,769,659.33 (1,286,808.74,2,432,375.2)	161.29 (118,220.25)	2,053,423.66 (1,584,338.44,2,524,971.56)	126.24 (96.01,154.8)	−1.13 (−1.27,-1)
Cyprus	1007.23 (849.82,1425.88)	126.08 (106.41,179.8)	1919.37 (1271.94,2313.86)	118.6 (80.22,143.11)	−0.12 (− 0.18,-0.06)
Democratic People’s Republic of Korea	31,069.99 (18,794.58,47,524.82)	145.77 (91.68,215.33)	36,083.96 (24,028.348274.92)	125.19 (85.32,164.26)	−0.41 (− 0.48,-0.35)
Georgia	10,103.04 (8965.14,13,446.38)	150.42 (132.85,200.54)	17,967.83 (14,429.36,22,063.81)	135.96 (110.9165.68)	2.21 (1.9,2.52)
India	699,838.79 (463,196.48,1,161,227.73)	80.1 (57.24,126.44)	1,013,246.94 (802,753.13,1,241,603.16)	77.08 (61.02,94.35)	−0.32 (− 0.42,-0.22)
Indonesia	141,953.13 (91,504.18,228,525.46)	81.2 (56.45,122.81)	201,930.16 (137,531.94,263,327.39)	79.72 (55.15,102.82)	−0.04 (− 0.11,0.02)
Iran (Islamic Republic of)	91,481.49 (49,513.61,123,035.58)	175.03 (102.02,223.69)	128,546.71 (67,681.65,153,605.56)	156.38 (81.96,187.04)	−0.31 (− 0.56,-0.07)
Iraq	30,232.26 (20,988.25,56,241.26)	195.88 (139.32,342.11)	79,864.69 (57,728.03,103,168.15)	225.65 (162.92,289.01)	0.64 (0.42,0.86)
Israel	7045.21 (6219.97,10,155.65)	146.3 (129.38,209.83)	15,795.59 (10,099.78,17,865.07)	158.24 (102.07,178.76)	0.12 (−0.08,0.33)
Japan	56,617.15 (52,505.08,77,096.08)	43.63 (40.3,60.52)	88,580.82 (44,446.44,105,611.21)	54.37 (29.52,63.5)	1.02 (0.87,1.18)
Jordan	3222.42 (2476.83,4376.6)	108.92 (85.37,150.66)	9737.27 (6955.98,12,041.62)	97.82 (69.49,120.61)	−0.45 (− 0.56,-0.34)
Kazakhstan	8425.2 (6499.68,14,258.93)	52.12 (39.74,90.38)	22,415.97 (11,163.62,29,143.67)	118.13 (58.55,153.11)	4.01 (3.29,4.73)
Kuwait	1042.23 (893.15,1339.11)	72.44 (62.78,94.78)	2477.72 (1736.02,3106.16)	66.18 (47.03,82.8)	0.22 (−0.02,0.47)
Kyrgyzstan	2538 (1930.23,4622.91)	61.47 (48.02,107.45)	5935.61 (3626.57329.93)	96.47 (57.76,119.86)	1.58 (1.42,1.75)
Lao People’s Democratic Republic	4875.24 (2002.29,10,819.99)	118.59 (57.39,238.3)	6168.85 (3908.77,8643)	94.87 (60.59,132)	−0.81 (− 0.85,-0.78)
Lebanon	3813.61 (2840.25215.41)	128.89 (98.43,173.78)	6093.85 (4252.08,7858.05)	117.25 (82.62,151.49)	−0.17 (− 0.28,-0.05)
Malaysia	11,972.5 (7140.83,15,390.55)	76.65 (47.57,98.24)	21,223.18 (13,292.23,28,837.73)	68.89 (43.62,93.06)	− 0.48 (− 0.72,-0.25)
Maldives	223.3 (121.5459.49)	109.53 (67,205.81)	343.73 (266.02,473.65)	77.32 (59.56,102.92)	−1.33 (−1.51,-1.16)
Mongolia	1348.81 (887.64,2776.24)	73.18 (48.71,142.22)	4144.72 (2890.59,5477.35)	127.14 (86.67,168.16)	2.53 (2.12,2.93)
Myanmar	42,044.07 (19,768.06,86,234)	105.72 (54.63,201.07)	50,000.4 (33,503.31,68,455.5)	93.51 (62.6128.41)	−0.54 (− 0.66,-0.42)
Nepal	17,122.5 (10,261.74,29,709.02)	78.93 (53.36,127.41)	19,582.43 (13,330.68,25,784.3)	68.94 (47.36,90.72)	−0.28 (− 0.44,-0.12)
Oman	1001.57 (736.59,1425.1)	65.38 (48.6,97.07)	2519.74 (1453.33280.19)	72.57 (43.78,91.34)	0.8 (0.54,1.07)
Pakistan	107,534.81 (69,104.32,170,537.13)	86.05 (62.68,124.58)	237,760.47 (161,751.61,309,213.33)	105.47 (72.77,137.31)	0.75 (0.66,0.83)
Palestine	4147.16 (2973.75,6255.71)	255.22 (190.39,372.37)	8903 (6939.05,11,030.84)	232 (175.63,279.46)	−0.35 (− 0.48,-0.22)
Philippines	54,877.17 (43,762.78,78,220.21)	96.71 (79.53,123.42)	90,815.42 (69,387.41,109,515.31)	86.56 (65.8104.73)	−0.44 (− 0.63,-0.25)
Qatar	317.44 (222.75,546.51)	107.22 (79.36,176.24)	1692.29 (1129.56,2800.78)	85.5 (61.1128.41)	−0.77 (− 0.89,-0.65)
Republic of Korea	43,710.22 (31,486.48,61,526.1)	108.12 (78.56,150.66)	46,150.05 (29,767.64,55,389.17)	71.43 (47.79,86.64)	−1.74 (−2.01,-1.47)
Saudi Arabia	7543.08 (4058.77,16,619.18)	60.02 (31.97,138.12)	26,453.41 (19,219.22,39,839.35)	79.41 (59.21,120.87)	1.21 (0.83,1.58)
Singapore	1728.69 (1534.75,2480.3)	64.08 (56.78,91.37)	3328.46 (1823.36,3953.17)	53.24 (29.26,63.75)	−0.54 (−0.8,-0.28)
Sri Lanka	8131.68 (6646.89,12,330.34)	52.55 (43.3,77.94)	17,071.33 (9487.08,24,055.38)	71.82 (40.58,100.76)	1.92 (1.57,2.27)
Syrian Arab Republic	15,173.08 (10,822.72,24,920.64)	155.29 (112.85,221.38)	20,391.06 (14,675.627540.06)	144.77 (104.67,194.09)	−0.36 (−0.51,-0.21)
Taiwan (Province of China)	16,820.57 (14,545.89,20,690.01)	87.85 (76.1107.56)	21,256.24 (13,293.32,28,521.95)	79.59 (48.66,104.1)	−0.63 (− 0.76,-0.49)
Tajikistan	9175.56 (6976.22,14,837.77)	203.64 (158,268.55)	18,572.03 (12,593.06,23,623.01)	216.7 (143.83,278.63)	−0.1 (− 0.3,0.11)
Thailand	51,376.66 (30,546.17,63,490.24)	98.28 (59.15,120.62)	81,091.52 (36,563.3118513.65)	99.54 (48.12,141.87)	−0.14 (− 0.36,0.08)
Timor-Leste	590.72 (287.75,1259.35)	79.59 (48.65,140.26)	1033.17 (520.54,1459.33)	86.93 (46,123.41)	0.31 (0.03,0.59)
Turkey	106,743.6 (48,327.82,162,996.19)	198.78 (92.75,303.49)	132,888.53 (63,487.74,183,729.62)	158.27 (76.68,215.47)	−0.61 (−0.94,-0.27)
Turkmenistan	2059.51 (1041.91,6218.48)	58.26 (30.6164.39)	7681.39 (5241.85,9913.45)	152.47 (103.55,196.33)	4.16 (3.42,4.91)
United Arab Emirates	2549.32 (1751.93518.56)	169.61 (119.31,250.26)	14,328.37 (8217.521136.76)	151.49 (91.5206.73)	−0.4 (−0.45,-0.34)
Uzbekistan	27,843.41 (24,505.64,35,187.04)	144.68 (126.53,177.47)	68,676.35 (44,839.62,85,680.91)	209.27 (139.09,259.09)	1.52 (1.39,1.64)
Viet Nam	25,810.55 (19,017.47,36,303.68)	44.13 (33.04,59.63)	64,970.32 (39,208.09,85,385.63)	64.89 (39.75,84.07)	1.9 (1.67,2.13)
Yemen	13,333.82 (5821.12,28,684.29)	107.04 (58.9206.86)	31,599.78 (18,697.52,46,414.79)	123.29 (74.87,181.54)	0.73 (0.61,0.84)

*DALYs* Disability-adjusted life years, *CI* Confidence interval, *EAPC* Estimated annual percentage changes, *UI* Uncertainty interval

### Changes in the burden in Asian divisions from 1990 to 2019

**Fig. 1:**
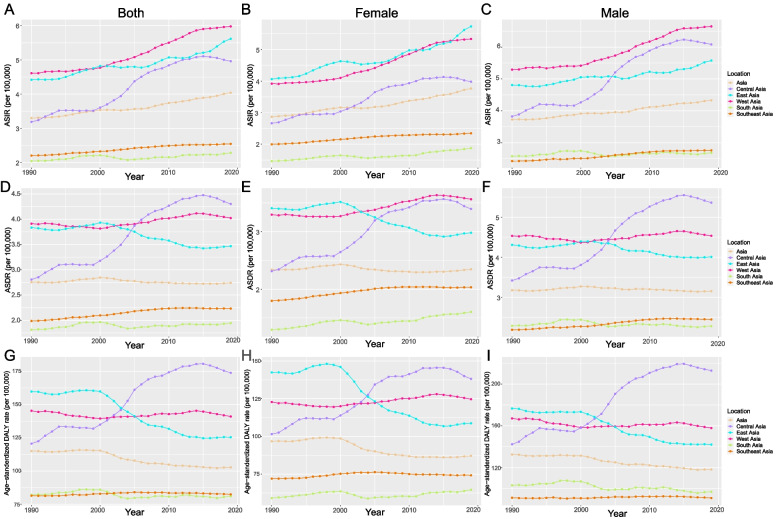
The change trends of the ASIR, ASDR, and the age-standardized DALY rate among different Asia divisions. Note: **A** indicated both ASIR, **B** indicated female ASIR, **C** indicated male ASIR, **D** indicated both ASDR, **E** indicated female ASDR, **F** indicated male ASDR, **G** indicated the age-standardized both DALY rates, **H** indicated the age-standardized female DALY rate, **I** indicated the age-standardized male DALY rate

**Fig. 2:**
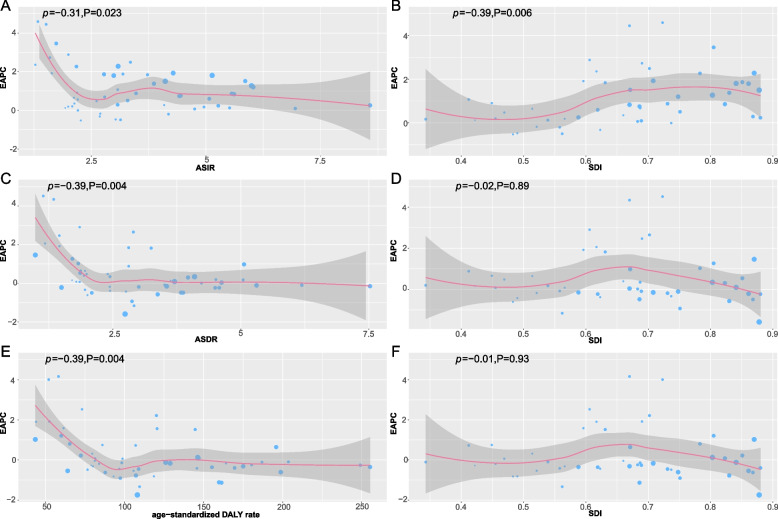
The correlation between EAPC and both ASR (incidence, death, and DALY) and SDI. Note: **A** indicated ASIR, **B** indicated the EAPC of ASIR, **C** indicated ASDR, **D** indicated the EAPC of ASDR, **E** indicated the age-standardized DALY rate, **F** indicated the EAPC of the age-standardized DALY rate. The circles represent countries that were available on SDI data. The size of the circle represents the number of CNS cancers patients and one circle represents a specific country. The ρ indices Pearson’s correlation coefficient and *P* values were derived from Pearson’s correlation analysis. ASR, age-standardized incidence/death/DALYs rate; EAPC, estimated annual percentage change; SDI, socio-demographic index

The ASIR increased in all Asia divisions, with Central Asia leading the way with an EAPC of 1.88 (95% CI, from 1.69–2.06) and South Asia having a gentle growth trend with an EAPC of 0.27 (95% CI, 0.18–0.35) (Table 1). The ASIR in West Asia was always at its highest level and had no obvious change (Fig. 1A). Moreover, EAPC showed a negative and weak correlation with both ASIR (*ρ* = − 0.31, *P* = 0.023, Fig. 2A) and SDI (*ρ* = − 0.39, *P* = 0.006, Fig. 2B). The ASDR was found to increase in most divisions besides in East Asia; Central Asia had the highest EAPC of 1.85 (95% CI, from 1.65 to 2.05) (Table 2). The ASDR in Central Asia soared in 2005 and has become the region with the highest ASDR, surpassing that in West Asia, which has been consistently high. Curiously, the ASDR in East Asia showed a downwards trend after 2000 (Fig. 1D). Furthermore, a negative correlation was found between EAPC and ASDR (*ρ* = − 0.39, *P* = 0.004, Fig. 2C), and a nonsignificant correlation was found between EAPC and SDI (*P* = 0.89, Fig. 2D). The age-standardized DALY rate increased in Asia overall. East Asia led the way with an EAPC of − 1.11 (95% CI, from − 1.25 to − 0.98), followed by South Asia and West Asia. In addition, Central Asia and Southeast Asia showed an increasing trend (Table 3). There was no significant difference between the age-standardized DALY rate and ASDR; they showed the same trend. However, the difference is that the value of West Asia is higher, and the value of East Asia is lower in the age-standardized DALY rate than in ASDR (Fig. 1G). A negative correlation was found between EAPC and the age-standardized DALY rate (*ρ* = − 0.39, *P* = 0.004, Fig. 2E), and a nonsignificant correlation was found between EAPC and SDI (*P* = 0.93, Fig. 2F).

### Changes in the burden in different countries of Asia from 1990 to 2019

**Fig. 3:**
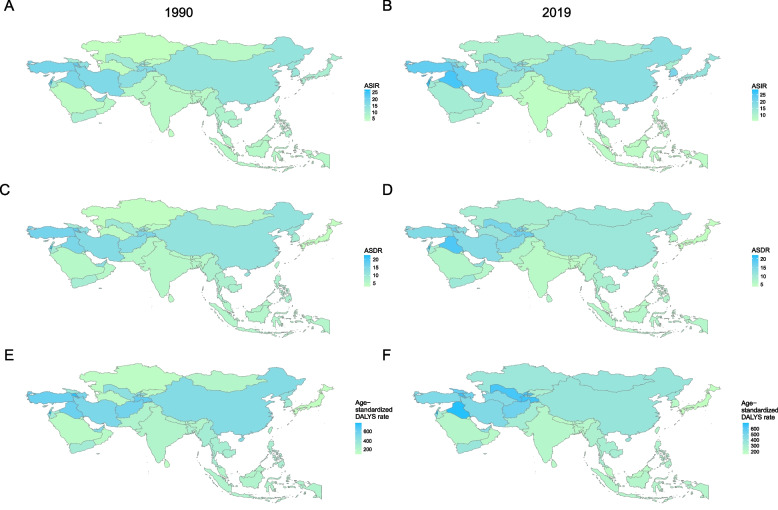
The spatial distribution of the ASIR, ASDR, and the age-standardized DALY rate in 1990 and 2019 in Asian countries

ASIR showed a rising trend in most countries. The increase was obvious in Kazakhstan and Turkmenistan, followed by Saudi Arabia and Mongolia, and mainly showed a downwards trend in Bangladesh, Maldives, and the Lao People’s Democratic Republic (Table 1). The top three countries with respect to ASIR were Palestine, Israel, and Iraq in 2019, and the countries with the lowest ASIR were Nepal, Bangladesh, and Vietnam (Fig. 3). Tajikistan, Turkey, and Israel had higher ASIRs among males than among females (Supplementary Fig. 1). The ASDR showed a downwards trend overall, and the Republic of Korea, Maldives, and Bahrain were the main countries with decreases. Nevertheless, the ASDR in Turkmenistan, Georgia, and Kazakhstan has increased substantially over the past 30 years. The top three countries with respect to ASDR were Palestine, Iraq, and Armenia in 2019, and the countries with the lowest ASDR were Japan, Singapore, and Bangladesh (Table 2 and Fig. 3). Over the past 30 years, the ASDR has continuously remained low in Nepal, Japan, and Singapore, with no appreciable changes (Supplementary Fig. 2). Among the GBD countries, the age-standardized DALY rate of Turkmenistan, Kazakhstan, and Mongolia was still on the obviously increasing, while other regions mostly were on the decline (Table 3). The countries with the highest age-standardized DALY rates in 2019 were Yemen, Vietnam, and Uzbekistan; the countries with the lowest age-standardized DALY rates were Singapore, Japan, and Kuwait (Fig. 3). Georgia and Turkmenistan had a noticeable change in the age-standardized DALY rate (Supplementary Fig. 3).

### Changes in the burden of CNS cancers in different sexes

Overall trends for both genders are comparable; however, after 2005, the ASIR among females increased considerably in East Asia, and the ASIR among males increased significantly in Central Asia. Furthermore, the ASIR for both males and females in West Asia is still increasing (Fig. 1B&C). In West and East Asia, the female ASDR is much greater than the male ASDR, and both have increased rapidly in Central Asia since 2000 (Fig. 1E&F). Before 2005, the age-standardized DALY rate in East Asia was much greater in females than in males, while in Central Asia, the age-standardized DALY rate in males followed a similar trajectory to the ASDR, both of which increased sharply after 2000 (Fig. 1H&I).

**Fig. 4:**
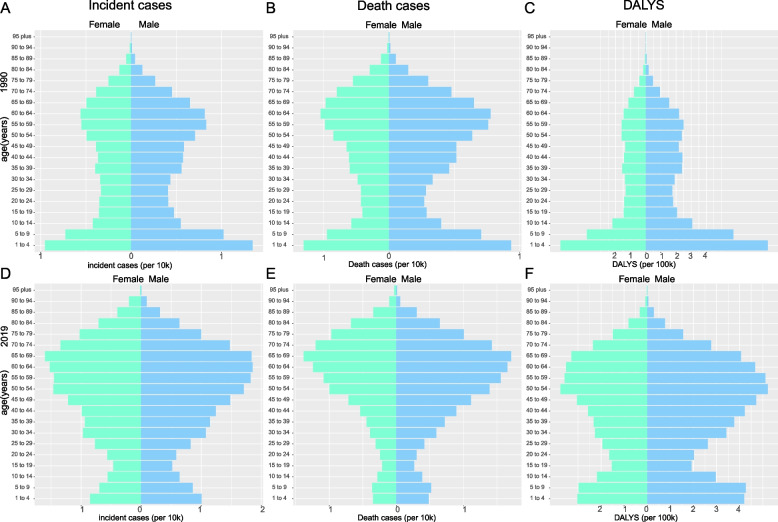
Comparison between age patterns and of the incidence (**A**), death cases (**B**), and DALYs (**C**) in 1990 and the incidence (**D**), death cases (**E**), and DALYs (**F**) in 2019 in Asia countries

In 1990, males and females sex ratio distributions for incidence, death cases, and DALYs were similar, with the highest ratio at 1–10 years old and decreasing thereafter and increasing at age 30. The only difference was that DALYs did not exhibit a statistically significant peak in 55–56-year-olds (Fig. 4A-C). Age trends in 2019 were comparable to those in 1990 for both sexes, with the highest risk group being 55 to 70 years old and a marked decline in morbidity and mortality between 1 and 10 years; however, DALYs were still higher in the 1–10 years old age range (Fig. 4D-F).

### Changes in the burden of CNS cancers at different ages

**Fig. 5:**
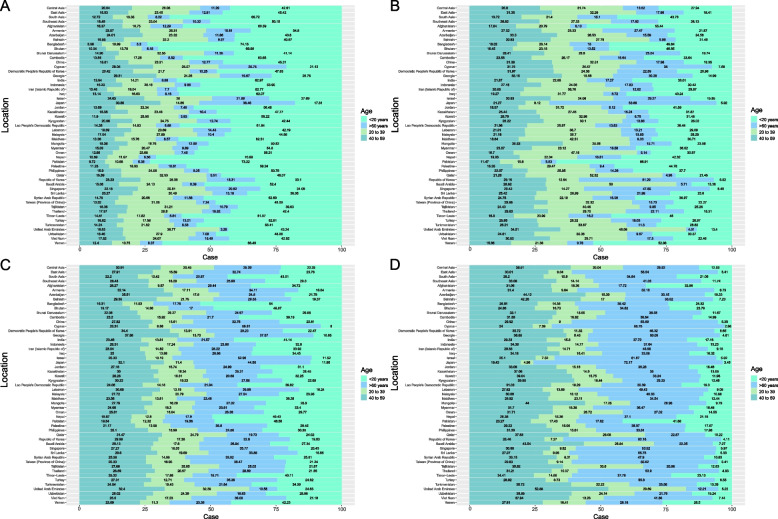
Comparison of the age-stratified proportions of incidence (**A**), and death cases (**C**) in 1990 and the incidence (**B**), and death cases (**D**) in 2019 in different Asian countries

South Asia had the highest number of incidences and deaths at < 20 years of age and the lowest number of incidences and deaths at > 60 years of age in 1990 (Fig. 5A&C). In 2019, South Asia still had the highest prevalence among those under the age of 20, while Central Asia had the lowest prevalence among those over 60 (Fig. 5B). Notably, deaths among those under 20 years old dramatically declined, especially in East Asia, which accounted for only 5.41%, while deaths were most frequent among those over 60 years of age (Fig. 5D).

In the age structure of the 1990 CNS cancers population, Bangladesh and Pakistan had the highest proportion of adolescent cases (more than 73%), whereas Japan had the highest proportion in the 40–59 and > 60 years old age groups. In addition, the smallest number of people were under the age of 20, accounting for only 17.81% of the overall number of cases (Fig. 5A). In most areas, the number of deaths in the 40–59 years and > 60 years age groups accounted for more than the number of cases, and the proportion of < 20-year-olds decreased (Fig. 5C). In contrast to 1990, the incidences of disease in those aged 40–59 and > 60 years increased, while the incidence decreased among individuals < 20 years old. Nonetheless, Japan continued to have the highest prevalence of patients > 60 years old and the lowest prevalence of patients < 20 years old (Fig. 5B). The most common age at death was > 60 years old, particularly in Japan (72.17%) (Fig. 5D).

### Predictions of CNS cancers incidence and death rates in Asia

**Fig. 6:**
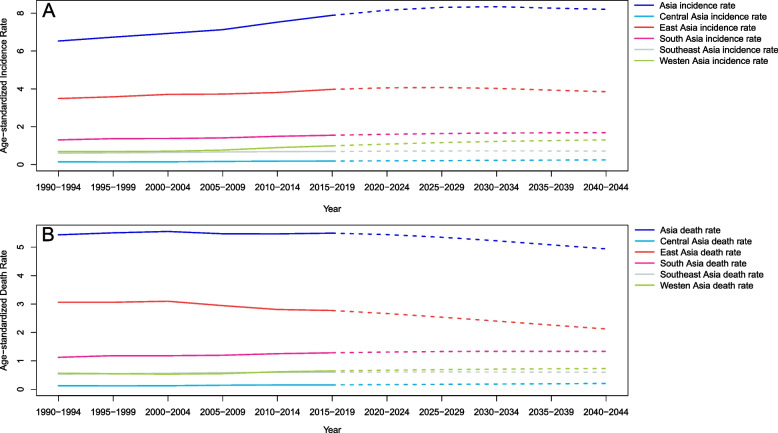
Trends in the age-standardized incidence (**A**) and deaths (**B**) rates of CNS cancers by different Asia divisions: observed (solid lines) and predicted rates (dashed lines)

The ASIR will continue to increase in Asia from 2020 to 2044, but the growth rate of each division is not obvious (Fig. 6A). While this is happening, the ASDR in Asia will gradually decrease, with East Asia experiencing the most pronounced reduction and no other noteworthy changes (Fig. 6B). More evident than the decrease in morbidity was the decrease in mortality (Fig. 6).

**Fig. 7:**
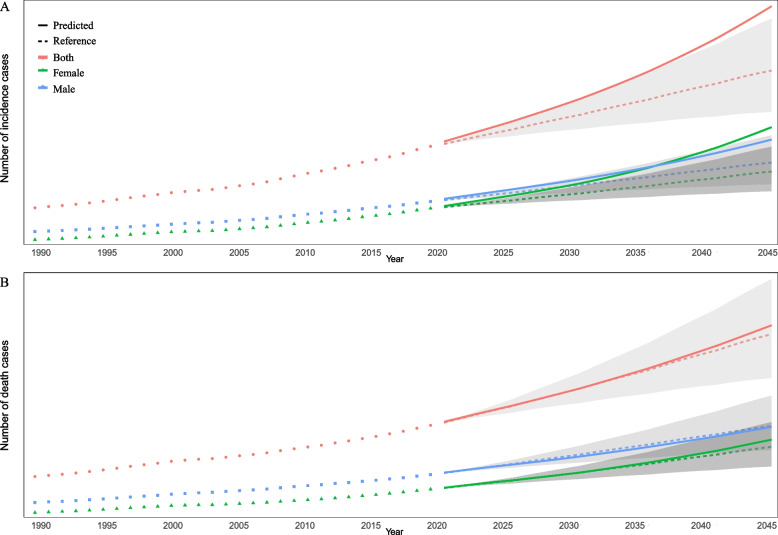
Trends in the number of incidences (**A**) and death (**B**) cases of CNS cancers by sex in Asia. Note: Solid lines indicated observed rates and dashed lines indicated predicted rates. Shading indicates if the rate remained stable (baseline reference), decreased by 1% per year (optimistic reference, lower limit), and increased by 1% per year (pessimistic reference, upper limit) based on the observed rate in 2019

From 2020 to 2044, both the incidence of and deaths from CNS cancers will continue to increase in Asia, especially among females. Our projections for both sexes are slightly higher than the negative reference of a 1% annual growth rate (Fig. 7). The incidence among females will increase from 92,506 in 2019 to 219,841, and the incidence among males will increase from 104,183 in 2019 to 200,282, both consistently above the baseline (Fig. 7A). The number of female deaths will increase from 90,603 in 2019 to 148,186, always above the baseline, and the number of male deaths will increase from 101,971 in 2019 to 162,581, always below the baseline (Fig. 7B).

## Discussion

Based on results from the GBD 2019 study, the current study showed that the global burden of CNS cancers has increased in the past 30 years, as demonstrated by an increase in incident cases, death, and DALYs. Even though the ASIR increased, ASDR and the age-standardized DALY rate showed a downwards trend (even though the change was not significant), which may be due to improvements in treatment and earlier, more precise diagnoses [2].

An in-depth analysis of the global burden of disease studies found that regional distribution, population growth, level of national development, age-specific incidence rates, changes in age structure, and gender may all be associated with cancers changes in incidence cases. Overall, the burden of CNS cancers increases significantly in Central Asia and decreases in East Asia. The reason for this may be that the level of development varies from region to region, with East Asia having a more favourable prognosis for treatment than other regions. It can therefore be inferred that DALYs associated with CNS cancers are negatively correlated with areas of lower national development capacity, which may be an indication of a lack of access to the highly specialized services required to treat some complicated diseases. These inequalities could cause delays in diagnosis and make it difficult to use available treatments in a way that would prevent or postpone death.

From a country perspective, Kazakhstan and Turkmenistan have the most significant CNS burden, while Japan and Singapore have the least. Age > 60 is the age group with the highest number of deaths, especially in Japan. While population growth was identified as the primary cause of the rise in overall cancers incidence in the low SDI quintile, ageing and changes in incidence rates played an equal (12% each) role in low-middle SDI countries, and population ageing was the primary cause of the rise in incidence in high-middle and high SDI countries [16]. Additionally, CNS cancers are given less importance in environments with limited resources due to its relative rarity compared to other cancers in adults. Therefore, differences in access to these services on various sociodemographic scales are accentuated. Our data indicate that age-standardized incidence rates are also at a higher level when socioeconomic development is high, but age-standardized mortality and DALY rates are reduced compared to low socioeconomic levels, which can be attributed to the level of care and more high-quality resources that result from higher SDI. This finding is in line with the trend towards improved CNS cancers survival seen by the Surveillance, Epidemiology, and End Results Program of the National Cancer Institute (relative 5-year survival probability from 26 years for those diagnosed from 8.20 to 1.20% for those diagnosed at 36 years) [17].

This study found that the highest number of deaths and DALYs was found in the 0–9 years age group in 1990. Monogenic genetic diseases and ionizing radiation are two primary risk factors for brain and other central nervous system malignancies in children, adolescents, and adults. In reality, radiation appears to be more carcinogenic in children – particularly younger children – with a definite dose–response association [18–20]. In contrast to 1990, incident cases increased for the 55–70 years age group, and incidence rates and deaths decreased significantly for the 1–10 years age group. Deaths among individuals under 20 years of age declined sharply, especially in East Asia, accounting for only 5.41% of all deaths. Mounting evidence from diverse studies suggests that higher socioeconomic position (SEP) is associated with an increased risk of adult CNS cancers compared to lower SEP individuals [21–25]. Therefore, the prevention of cancers of the brain and central nervous system in populations of high socioeconomic position should be emphasized.

We predicted that the ASIR would continue to increase in Asia from 2020 to 2044, but the ASDR will gradually diminish, with East Asia experiencing the most pronounced reduction. However, the number of cases and deaths related to CNS cancers in Asia is anticipated to increase as a result of population expansion and ageing, especially among females. The explanation for this could be due to the varied types of CNS cancers to which males and females are susceptible. Some studies have shown that the only neurological disorders that differ by less than 10% between males and females in terms of mortality and DALY rates are Alzheimer’s disease and other dementias, whereas only meningitis and epilepsy differ in terms of prevalence [17]. Policy-makers can use these predictive data to better avoid the emergence of cancers and enhance prognosis outcomes.

It is also crucial to keep in mind that these populations may experience a wide range of environmental exposures and influences. The only risk variables with reliable evidence are the positive correlation with ionizing radiation and the negative association with atopic illness [26–30]. A causal association has not been substantiated by thorough investigations of the relative influence of numerous other epidemiological risk variables in the population. Further research is required to determine how much environmental factors affect regional variations in incidence rates.

This study provided information on the prevalence of CNS cancers in Asia and investigated the relationships between incidence, death, DALY, and different demographic parameters. Forecast data for CNS from 2020 to 2044 are also provided. Policy-makers and governments require country-specific information on the global burden of different diseases to adjust their national benchmarks, implement relevant measures to reduce disease incidence, and allocate limited resources in their health care systems. Considering that the existing data in many countries are of low accuracy or missing, GBD research results can be used as a reference for studying the trends of different diseases in their respective locations.

There are several limitations in the study. Firstly, the uncertainty of GBD estimation, which results from the lack of actual illness burden data, is an unavoidable limitation. In this analytical mode, it is also inevitable that there would be differences in the data obtained using various data extraction techniques and in the veracity of various studies. Secondly, instead of the actual change in the age rate of a certain age, the fluctuations in incidence and mortality may be partially represented in the detection deviation linked to the modification in the screening system. Thirdly, data collected by cancer-related departments in various countries may have varied diagnosis standards, causing confusion. Finally, the forecast of CNS cancers for the next 25 years is influenced by a range of factors, many of which are unknown in the future; thus, numerous uncertainties may have an impact on the forecast results. We do not influence these intricate factors. Nonetheless, the findings of this study have been quite informative.

## Conclusions

Although the age-standardized death and DALY rates of CNS cancers have been declining, the number of cases, ASIR, deaths, and DALYs from CNS cancers have increased in Asia over the past 30 years. In contrast to 1990, the caseload has increased among the 55–70 years age group; the number of deaths among individuals younger than 20 years of age declined sharply, especially in East Asia, accounting for only 5.41% of all deaths; and the age group with the highest mortality rate was > 60 years, especially in Japan. The ASIR will continue to increase in Asia from 2020 to 2044, but the Asian ASDR will gradually diminish, with East Asia experiencing the most pronounced reduction. Nonetheless, the numbers of cases and deaths related to CNS cancers in Asia are anticipated to increase because of population expansion and ageing, especially among females. Taking these differences into consideration, health authorities and policy-makers should make better use of limited resources and formulate policies and measures.

### Supplementary Information

**Additional file 1.**

## Data Availability

To download the data used in these analyses, please visit the Global Health Data Exchange GBD 2019 data-input sources tool at http://ghdx.healthdata.org/gbd-2019/data-input-sources. No permission is required for anyone to access this data.
