# Supplementary material for: The burden of brain and central nervous system cancers in Asia from 1990 to 2019 and its predicted level in the next twenty-five years: Burden and prediction model of CNS cancers in Asia
Source: BMC Public Health. 2023 Dec 16;23:2522. doi: 10.1186/s12889-023-17467-w (PMC10724911; doi:10.1186/s12889-023-17467-w)

Legend of Supplementary Figure

Supplementary Figure 1 Changing trends in ASIR for male and female across 48 countries in Asia from 1990 to 2019

Supplementary Figure 2 Changing trends in ASDR for male and female across 48 countries in Asia from 1990 to 2019

Supplementary Figure 3 Changing trends in the age-standardized DALY rate for male and female across 48 countries in Asia from 1990 to 2019

Supplementary Figure 1 Changing trends in ASIR for male and female across 48 countries in Asia from 1990 to 2019

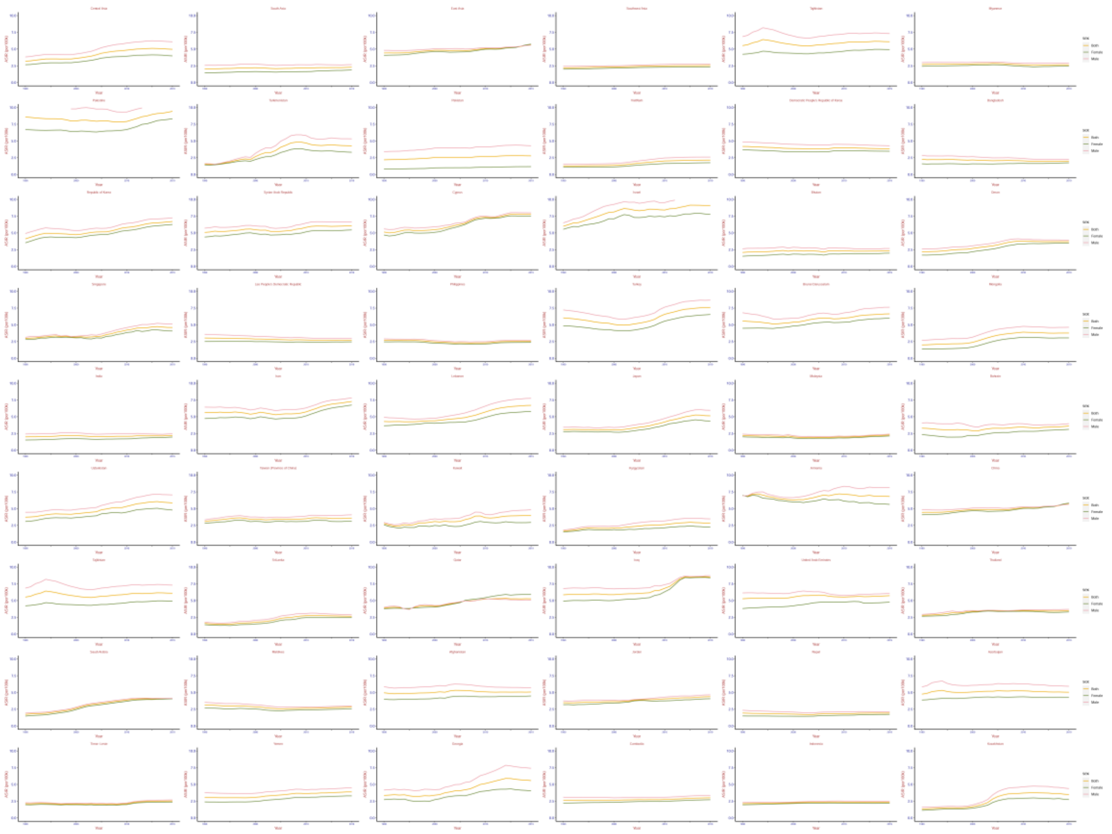

Supplementary Figure 2 Changing trends in ASDR for male and female across 48 countries in Asia from 1990 to 2019

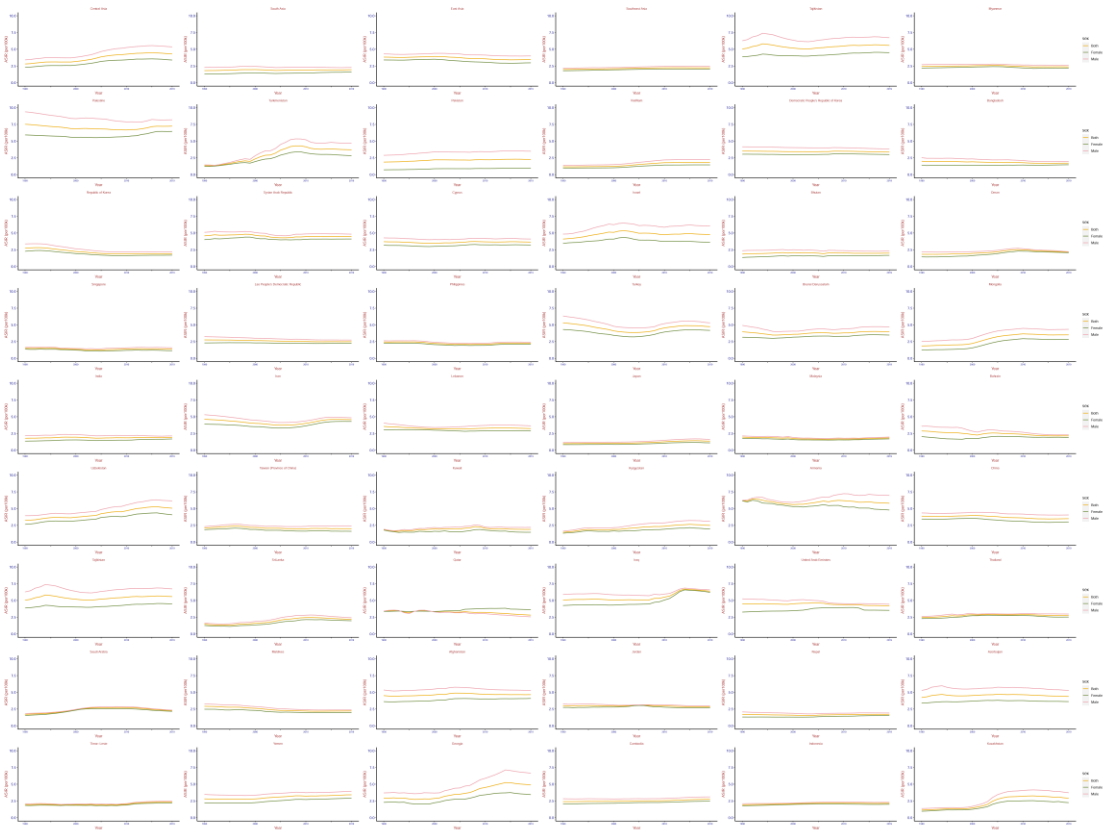

Supplementary Figure 3 Changing trends in the age-standardized DALY rate for male and female across 48 countries in Asia from 1990 to 2019

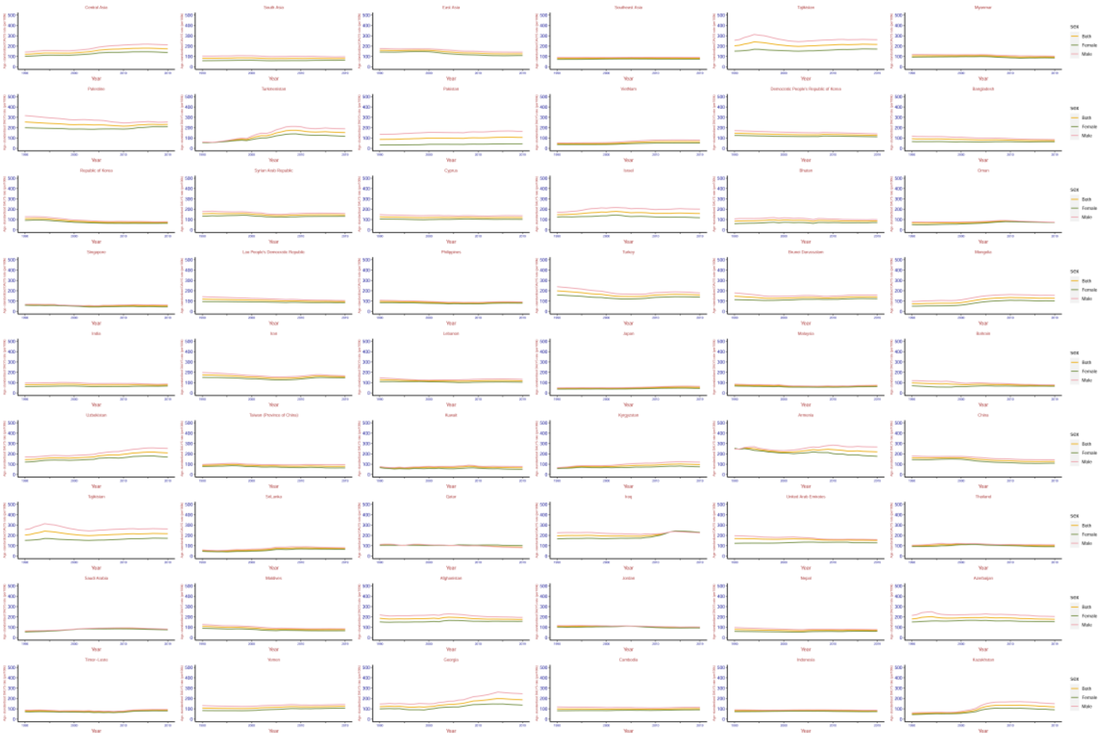

Supplement: Supplementary file 1 — Additional file 1. [file 12889_2023_17467_MOESM1_ESM.pdf]
